# Application of GelGreen™ in Cesium Chloride Density Gradients for DNA-Stable Isotope Probing Experiments

**DOI:** 10.1371/journal.pone.0169554

**Published:** 2017-01-05

**Authors:** Jingfeng Gao, Kailing Pan, Hongyu Li, Xiaoyan Fan, Lixin Sun, Shujun Zhang, Yongqing Gao

**Affiliations:** 1 National Engineering Laboratory for Advanced Municipal Wastewater Treatment and Reuse Technology, College of Environmental and Energy Engineering, Beijing University of Technology, Beijing, China; 2 Research and Development Center of Beijing Drainage Group Co., Ltd., Beijing, China; German Cancer Research Center, GERMANY

## Abstract

In this study, GelGreen^™^ was investigated as a replacement for SYBR^®^ Safe to stain DNA in cesium chloride (CsCl) density gradients for DNA-stable isotope probing (SIP) experiments. Using environmental DNA, the usage of GelGreen^™^ was optimized for sensitivity compared to SYBR^®^ Safe, its optimal concentration, detection limit for environmental DNA and its application in environmental DNA-SIP assay. Results showed that GelGreen^™^ was more sensitive than SYBR^®^ Safe, while the optimal dosage (15X concentration) needed was approximately one-third of SYBR^®^ Safe, suggesting that its sensitivity was three times more superior than SYBR^®^ Safe. At these optimal parameters, the detection limit of GelGreen^™^-stained environmental DNA was as low as 0.2 μg, but the usage of 0.5 μg environmental DNA was recommended to produce a more consistent DNA band. In addition, a modified needle extraction procedure was developed to withdraw DNA effectively by fractionating CsCl density gradients into four or five fractions. The successful application of GelGreen^™^ staining with ^13^C-labeled DNA from enriched activated sludge suggests that this stain was an excellent alternative of SYBR^®^ Safe in CsCl density gradients for DNA-SIP assays.

## Introduction

Stable isotope probing (SIP) is a technique that links microbial identity to metabolic function, and has proven to be a useful tool in microbial ecology. It works by incubating environmental samples with stable isotope labeled substrates (e.g. ^13^C) that when consumed are incorporated into the targeted biomarker molecules, such as phospholipid fatty acid (PLFA) [1], DNA [2] and RNA [3]. Then, by isopycnic density gradient centrifugation, the labeled molecules can be separated and used for other downstream analyses. Application of PLFA-SIP however is limited by the lack of knowledge of the profiles of the targeted molecules, especially in most uncultured microorganisms [4]. This limitation makes it difficult to confirm the phylogenetic information of the labeled microorganisms. For RNA-SIP on the other hand, the downstream applications are limited by low yields of mRNA retrieved from environmental samples and their incomplete separation after ultracentrifugation [5]. In contrast, DNA-SIP is remarkable with its ability to provide insights on metabolic functions of active microorganisms and their phylogenetic information at the same time. Further, stable isotope labeled (‘heavy’) DNA can be obtained after short-term incubation and separated from the unlabeled (‘light’) DNA by isopycnic centrifugation. DNA-SIP combined with sequence-based approaches, such as Sanger sequencing [6], high-throughput sequencing [7] and metagenomics [8], brings new insights into the genetic basis of functional information of microorganisms. Inarguably, DNA-SIP has become the most popular method being used to investigate the active microbes in different habitats.

DNA-SIP technique is mainly composed of four main steps, which starts by (1) incubating environmental samples with stable isotope labeled compounds; then, (2) extracting DNA from the incubated samples and then separating by ultracentrifugation, followed by (3) identifying ‘heavy’ DNA; and the (4) downstream analyses of the ‘heavy’ DNA. The key step, which is the identification of the ‘heavy’ DNA, can be carried out using a number of approaches such as nucleic acid staining [2, 9], isotope ratio mass spectrometry (IRMS) analysis [3], fingerprinting analysis [10–12] and quantitative real-time PCR (qPCR) [5]. The most popular however is the staining technique because of its convenience and non-complex operational costs [13, 14], which usually uses the mutagenic ethidium bromide (EtBr) [2] and SYBR^®^ Safe [9]. However, because of low sensitivity of EtBr staining, a lot of DNA (at least 15 μg) is required to generate a clear band visualized by UV illumination [5]. Further, UV exposure may also cause further DNA mutation, which could influence the follow-up analyses. Although safer as a weak mutagen and has higher sensitivity than EtBr, SYBR^®^ Safe still requires at least 0.5 μg pure culture DNA to form a clear band [9]. This has significant implications to microbial studies since DNA extracted from natural or environmental samples are usually lower in concentrations and form a more dispersed band than those from pure culture after ultracentrifugation. Recently, a new generation of fluorescent nucleic acid stain has been gaining more attention, called GelGreen^™^, which has lower toxicity, higher sensitivity and exceptional stability. As an alternative, GelGreen^™^ is 2–3 times more sensitive than SYBR^®^ Safe and can detect 0.5 ng DNA in agarose gel [15]. To date however, no studies yet have reported the application of GelGreen^™^ to DNA-SIP, particularly in cesium chloride (CsCl) density gradients.

Following the identification of the labelled DNA, retrieval after ultracentrifugation is a prerequisite for follow-up analyses, which can be performed by fractionating the complete gradients (traditional fractionation) [3] or by directly retrieving the DNA bands using needles and syringes (needle extraction) [2]. For traditional fractionation, syringe pump is used to pump water into the top of ultracentrifuge tube in a precisely controlled flow rate and the fractions are collected from the bottom of the tube. However, disruption of gradients sometimes cannot be avoided when syringe pump delivers water as displacement. This method is usually combined with qPCR to quantify all the fractions to find isotope labelled DNA [5]. Usually, CsCl density gradients in an ultracentrifuge tube are divided into 15–16 fractions [7, 16], making it very time consuming and complicated. Meanwhile, needle extraction is combined with DNA staining making it faster and more visual. However, in the process of retrieving the target DNA bands, the overlying non-target layers may disrupt the target DNA bands. So far, no satisfactory methods have been developed to retrieve DNA from CsCl gradients after ultracentrifugation.

In this study, we developed a protocol using GelGreen^™^ staining in CsCl density gradients applied to environmental DNA. Based on the positions of DNA bands, a modified needle extraction procedure was designed to fractionate the total gradients. Furthermore, DNA extracted from enriched activated sludge exposed to ^13^C-labeled substrate was used to evaluate the potential of GelGreen^™^ staining in CsCl density gradients for environmental DNA-SIP assay. Using qPCR technique, bacterial 16S rRNA gene was quantified to examine the quantities of total DNA in fractions, while the *amoA* gene of ammonia oxidizing archaea (AOA) and ammonia oxidizing bacteria (AOB) were selected to gain insights into the abundance of functional aspects of ammonia oxidizing microorganisms. The modified needle extraction procedure provided a new and simple way to accurately retrieve DNA, saving time and cost of follow-up analyses.

## Materials and Methods

### DNA extraction

Activated sludge was collected from Gaobeidian wastewater treatment plant (WWTP) in Beijing and then was freeze-dried by Labconco Freezone 1 L (Labconco, USA). This study was approved by the Research and Development Center of Beijing Drainage Group Co., Ltd., and no endangered or protected species were involved in the WWTP. The dried sludge was used to extract DNA using FastDNA SPIN kit for soil (Qiagen, CA, USA) following the manufacturer’s instructions. The concentration of extracted DNA was determined using Nanodrop Spectrophotometer ND-1000 (Thermo Fisher Scientific, USA) and stored at –20°C until further analyses.

### Target DNA staining and CsCl density gradient ultracentrifugation

**Fig 1:**
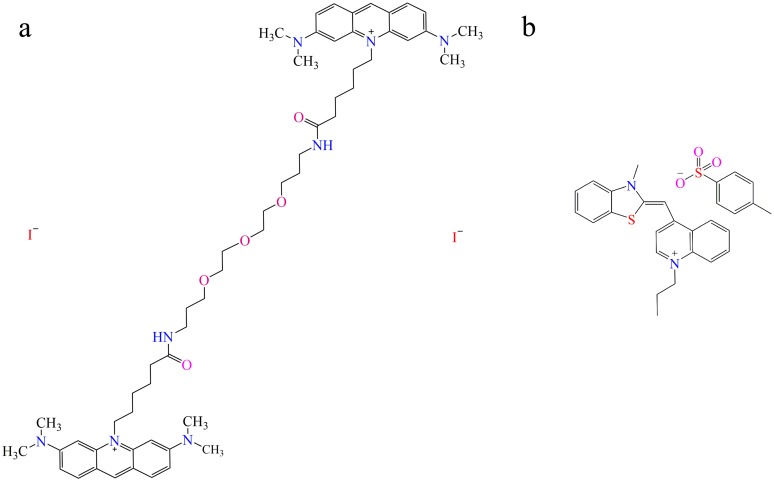
Molecular structures of GelGreen^™^ (a) and SYBR^®^ Safe (b).

To compare sensitivity, both GelGreen^™^ (Biotium, USA) and SYBR^®^ Safe (Invitrogen, USA) were used to stain DNA in CsCl density gradients. The molecular formulas of GelGreen^™^ and SYBR^®^ Safe are C_56_H_80_I_2_N_8_O_5_ and C_28_H_28_N_2_O_3_S_2_, and their molecular structures are shown in Fig 1a and 1b, respectively. In addition, separate CsCl density gradients made up of TE or GB buffer were compared to determine the more optimal conditions for GelGreen^™^ staining. Modified from previous studies [9, 17], the gradient medium containing GelGreen^™^ was prepared as follows: a final volume of 1mL gradient was prepared by adding 5 μg DNA mixed with TE buffer (10 mM Tris-HCl (pH = 8), 1.0 mM EDTA (pH = 8)) or GB buffer (0.1 M Tris-HCl (pH = 8), 0.1 M KCl, 1.0 mM EDTA) and then 1 g CsCl powder was added. After gentle mixing, 4.7 mL of 1 g mL^−1^ CsCl solution and 8.85 μL 10000X GelGreen^™^ (final concentration was 15X) were added into the mixture. Moreover, the optimal concentration of GelGreen^™^ was also tested by setting different final concentrations at 1X, 5X, 10X, 15X, 20X, 25X, 30X and 50X. For each treatment, the dosage of environmental DNA was 5 μg and TE buffer was used. In addition, the detection limit for environmental DNA was also investigated by adding 0 μg, 0.1 μg, 0.2 μg, 0.5 μg, 0.8 μg, 1.0 μg, 2.0 μg and 5.0 μg DNA separately into CsCl density gradients with 15X GelGreen^™^, stabilized by TE buffer.

The density of the gradient medium was measured by AR200 digital hand-held refractometer (Reichert, USA) and the final density was adjusted to 1.72 g mL^−1^. For the gradient medium containing SYBR^®^ Safe, the preparation was the same as above except that the volume of 10000X SYBR^®^ Safe was 25 μL (final concentration was approximately 43X) [9].

The gradient media were loaded into 5.1 mL polyallomer Quick-Seal centrifuge tubes and centrifuged at 190,000 x *g* for 44 hours at 20°C with vacuum, maximum acceleration and without brake using L8-70M ultracentrifuge (Beckman, USA). After ultracentrifugation, DNA bands were detected by illuminating with blue light using the Dark Reader Handheld Lamp (Clare Chemical Research, USA).

### DNA retrieval and purification

**Fig 2:**
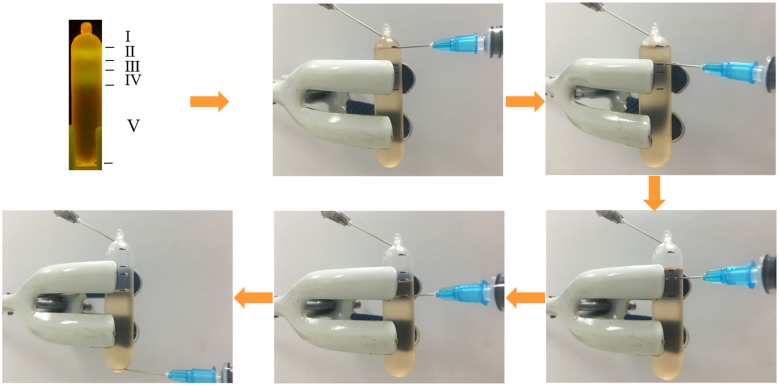
Schematic diagram summarizing steps of modified needle extraction. Fractions I, II, III, IV and V show the range of fractions withdrawn by needles and syringes.

A modified needle extraction procedure was developed to fractionate all the gradients, and the details are shown in Fig 2. Based on the location of the ‘light’ and ‘heavy’ DNA bands, the all gradients in the centrifuge tube were divided into five fractions, here referred to as fractions I, II, III, IV and V from top to bottom, respectively. The ‘light’ and ‘heavy’ DNA were in fractions II and IV, respectively, which were marked on the tube. Retrieval of DNA was performed by starting from the top going to the bottom. The needle and syringe were inserted into the bottom of fraction I, and then the gradient was withdrawn gently and carefully. The same technique was applied individually for each of the remaining layers until all were withdrawn. If only ‘light’ DNA existed, the gradients were divided into fractions I, II, III and IV from top to bottom and ‘light’ DNA was in fraction II. The gradients below fraction II were divided into fractions III and IV because the liquid was too much for one fraction.

Two volumes of PEG solution were used to precipitate DNA from the fractions, followed by centrifugation to collect precipitates. After discarding the supernatant, 500 μL of 70% ethanol was added to wash the pellets and then dissolved in 30 μL TE buffer.

### DNA-SIP incubation with enriched activated sludge

DNA-SIP incubation with ^13^C-labeled synthetic wastewater was performed in 500 mL batch reactors in triplicate. A total of 200 mL enriched activated sludge collected from a nitrifying lab-scale sequencing batch reactor was inoculated to each incubation. The ^13^C-labeled synthetic wastewater contained 0.214 g L^−1^ Na_2_^13^CO_3_, 0.107 g L^−1^ NH_4_Cl, 0.585 g L^−1^ NaCl, 0.054 g L^−1^ KH_2_PO_4_, 0.075 g L^−1^ KCl, 0.147 g L^−1^ CaCl_2_ 2H_2_O, 0.049 g L^−1^ MgSO_4_ 7H_2_O [18], and 1mL non-chelated trace element mixture [19]. Mixed liquor suspended solids (MLSS) was controlled at 5000 mg L^−1^. The incubations were conducted at pH = 7.8, DO > 2 mg L^−1^ and shaken at 100 r min^−1^ in dark at 30°C. The concentration of ammonia in supernatant was measured every 30 min. When the concentration of ammonia reached close to zero, incubation was extended for another cycle before being ended. Microcosms were sampled at 20 cycles and then freeze-dried using Labconco Freezone 1L (Labconco, America). The dry sludge samples from the three replicates were pooled as one sample. After DNA extraction, 5 μg and 10 μg DNA were added into CsCl density gradient with 15X GelGreen^™^, respectively. And then the media were processed to CsCl density gradient ultracentrifugation as mentioned above.

### QPCR for DNA in fractions

To verify the accuracy of GelGreen^™^ staining technique, quantities of DNA in fractions were estimated by qPCR with bacterial 16S rRNA gene primers (1055F (5'-ATGGCTGTCGTCAGCT-3') and 1392R (5'-ACGGGCGGTGTGTAC-3')) [20]. The 20 μL reaction mixture contained 10 μL GoTaq^®^ qPCR Master Mix (Promega, USA), 0.2 μM of each primer, and 2 μL of template DNA. Amplification reaction conditions were as follows: 95°C for 10 min, 40 cycles of 95°C for 30 s, 53°C for 1 min, and 72°C for 1 min [20]. Plasmid was obtained from the positive colony containing bacterial 16S rRNA gene, which was used to construct standard curve with dilution from 2.2×10^8^ to 2.2×10^1^ copies. All standard plasmids and samples were performed in triplicate. The correlation coefficients (*R*^2^) and efficiencies of standard curves were 0.994–0.995 and 95.8–97.1%, respectively.

### QPCR for *amoA* gene of AOA and AOB

The copy numbers of AOA *amoA* gene in fractions were also investigated by qPCR using the primer sets GenAOAF (5'-ATAGAGCCTCAAGTAGGAAAGTTCTA-3') and GenAOAR (5'-CCAAGCGGCCATCCAGCTGTATGTCC-3') [21]. Primers amoA-1Fmod (5'-CTGGGGTTTCTACTGGTGGTC-3') and GenAOBR (5'-GCAGTGATCATCCAGTTGCG-3') were utilized to quantify the abundance of AOB *amoA* gene in the different fractions [21]. For AOA, the reaction mixture contained 10 μL GoTaq^®^ qPCR Master Mix (Promega, USA), 0.2 μM of each primer, and 2 μL of template DNA. And the qPCR conditions were 95°C for 10 min, 40 cycles of 95°C for 30 s, 56°C for 1 min, and 72°C for 1 min. For AOB however, the annealing temperature used was 58°C. The standard curves were generated by 10-fold serial dilutions of standard plasmids. For AOA, correlation coefficient (*R*^2^) and efficiency of standard curve were 0.997 and 85.1%, respectively. For AOB, correlation coefficient (*R*^2^) and efficiency of standard curve were 0.997 and 100%, respectively.

## Results

### Comparison of GelGreen^™^ and SYBR^®^ Safe staining in CsCl density gradients with TE or GB buffer

**Fig 3:**
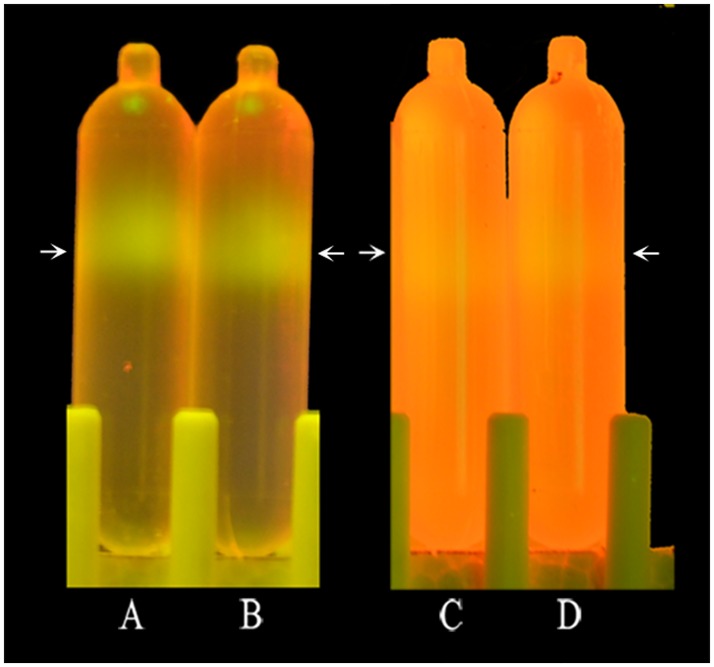
Comparison of GelGreen^™^ and SYBR^®^ Safe staining with 5 μg environmental DNA in CsCl density gradients with TE or GB buffer. A, B, C and D treatment represent GelGreen^™^ in TE buffer, GelGreen^™^ in GB buffer, SYBR^®^ Safe in TE buffer and SYBR^®^ Safe in GB buffer, respectively. The locations of DNA bands are pointed by arrows.

The ‘light’ and ‘heavy’ DNA were separated based on atomic density and both stained well by nucleic acid stains. Thus, the environmental DNA (‘light’ DNA) was directly used in this protocol. Previous studies [9, 17] showed that both TE and GB buffer are suitable to be used in CsCl gradient to balance buoyant density and protect DNA. However, it is unknown which buffer is more suitable for both GelGreen^™^ and SYBR^®^ Safe staining. Fig 3 shows the results of ultracentrifugation with 5 μg environmental DNA individually stained by GelGreen^™^ or SYBR^®^ Safe in CsCl-TE or CsCl-GB medium. The added GelGreen^™^ volume of 8.85 μL (final concentration was 15X) was determined by preliminary experiments, while the volume of SYBR^®^ Safe used was 25 μL (final concentration was approximately 43X) which was the optimal dosage reported by Martineau *et al*. [9].

Because environmental DNA without ^13^C-labeled incubation was used, only one DNA band was visible in each ultracentrifuge tube. As shown in Fig 3, DNA bands were all visible in the upper layers of the centrifuge tubes. The DNA bands stained by GelGreen^™^ occurred in the same layers as those stained by SYBR^®^ Safe, indicating consistency. Regardless of the buffer used, the DNA bands stained by GelGreen^™^ were always stronger than those stained by SYBR^®^ Safe. In addition, the needed dosage for optimal staining by SYBR^®^ Safe was 1.8 times larger than GelGreen^™^, suggesting that the latter has higher sensitivity. Tubes stained with SYBR^®^ Safe also had more background fluorescence associated with the dye itself, which was so strong that shielded the fluorescence emitted by DNA bands. On the other hand, GelGreen^™^-stained samples had less background noise producing more defined DNA bands. Since no significant difference was observed between the two dyes, the TE buffer was preferred for subsequent applications due to its simpler composition.

### The effect of GelGreen^™^ concentration on CsCl density gradient staining

**Fig 4:**
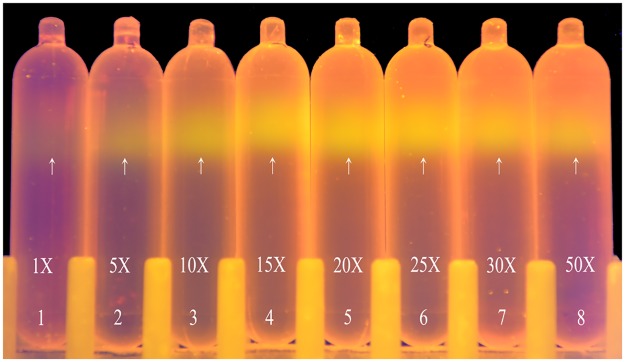
Optimization of GelGreen^™^ concentration in CsCl density gradients. The concentrations of GelGreen^™^ (from left to right) were 1X, 5X, 10X, 15X, 20X, 25X, 30X and 50X in CsCl density gradients, respectively. The locations of DNA bands are pointed by arrows.

Fig 4 illustrates the results of CsCl density gradient ultracentrifugation with different concentrations of GelGreen^™^ dye. The locations of DNA bands did not vary as the concentration of GelGreen^™^ changed, suggesting the stability of GelGreen^™^ staining. The fluorescence intensities of DNA bands strengthened with the GelGreen^™^ concentration increasing from 1X to 15X but saturation was observed for concentrations higher than 15X. The excess GelGreen^™^ precipitated out of the solution after ultracentrifugation that accumulated at the top of centrifuge tube and emitted strong background fluorescence that shaded the fluorescence signals from the DNA. From 20X to 50X, DNA fluorescence slightly weakened with increasing concentration of GelGreen^™^. Based on these results, 15X was determined to be the optimal GelGreen^™^ concentration for staining DNA in CsCl density gradients.

### Detection limit of environmental DNA in CsCl density gradients stained by GelGreen^™^

**Fig 5:**
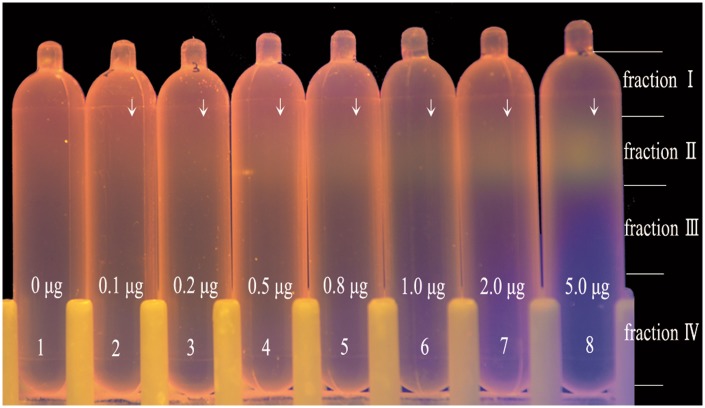
Detection limit of environmental DNA in CsCl density gradients stained by GelGreen^™^ under its optimal concentration. From left to right, 0 μg, 0.1 μg, 0.2 μg, 0.5 μg, 0.8 μg, 1.0 μg, 2.0 μg and 5.0 μg environmental DNA were used. The locations of DNA bands are indicated by arrows. Fractions I, II, III and IV show the range of fractions withdrawn by needles and syringes.

The fluorescence intensities of DNA bands were positively correlated with DNA dosage (Fig 5). Although the location of 0.1 μg DNA band was significantly brighter than that in the control tube (0 μg DNA), it was still difficult to exactly recognize the DNA band. Using 0.2 μg environmental DNA, the fluorescence intensity of the DNA band was still weak but identifiable. It was at 0.5 μg dosage when the DNA band became easily detected. These results suggest that 0.5 μg environmental DNA was needed to produce a detectable band in a CsCl density gradient stained.

### Quantitative analysis of total DNA in gradient fractions

**Fig 6:**
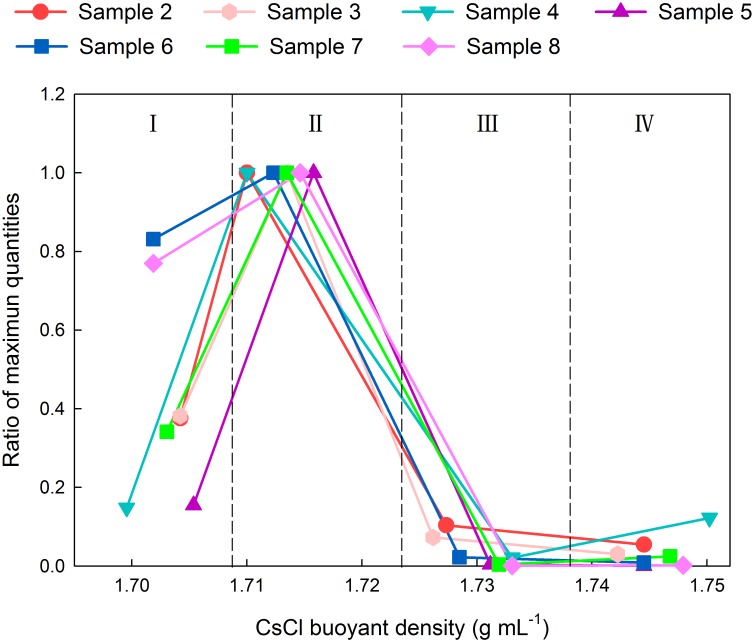
Quantitative distribution of bacterial 16S rRNA gene copy number across all the fractions.

Ratios of the copy number of bacterial 16S rRNA gene in each fraction relative to the maximum copy number from the same ultracentrifuge tube are shown in Fig 6. The peaks of maximum ratios all appeared in fractions II of each tested sample, which were consistent with the results of the visualization of DNA bands. Fewer copies of the gene were found in fractions III and IV compared to fraction I.

### Application of GelGreen™ in CsCl density gradients for environmental DNA-SIP assay

**Fig 7:**
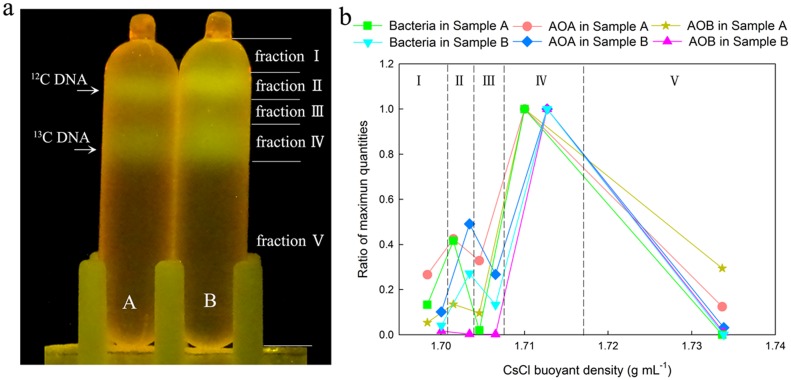
Visualization of DNA bands stained by GelGreen^™^ with environmental DNA after SIP incubation (a) and relative abundance of bacterial 16S rRNA gene, *amoA* gene of AOA and AOB (b). In Fig 7a, 5 μg and 10 μg labeled-DNA were added into samples A and B, respectively. Fractions I, II, III, IV and V show the range of fractions fractionated by needles and syringes.

The dosage of labeled DNA, extracted from enriched activated sludge incubated with special substrate, was also investigated by GelGreen^™^ staining in CsCl density gradients. As shown in Fig 7a, two separated DNA bands were easily detected in 5 μg and 10 μg labeled-DNA (samples A and B), which corresponded to ^12^C-DNA and ^13^C-DNA, respectively. In sample A, both ^12^C-DNA and ^13^C-DNA bands were clearly visible, with fluorescence intensity of ^13^C-DNA band stronger than the ^12^C-DNA band. This indicates that GelGreen^™^ was applicable in staining DNA for environmental SIP assays. Compared to sample A, the fluorescence intensities of DNA bands in sample B were stronger, suggesting that increased dosage of DNA could strengthen the effect of GelGreen^™^ staining.

As shown in Fig 7a, based on the location of ^12^C-DNA and ^13^C-DNA bands, the gradients from each centrifuge tube were fractionated into fractions I to V from top to bottom, respectively. Fig 7b shows the relative abundance of bacterial 16S rRNA gene, AOA and AOB *amoA* genes in the different fractions from samples A and B. For both samples A and B, peaks of the relative abundance of the 16S rRNA gene occurred in fractions II and IV, and the highest copy numbers appeared in fraction IV. For sample A, two peaks appeared in the relative abundance curve of AOA *amoA* gene. The maximum value of AOA occurred in fraction IV, which was characterized as a heavy fraction. The relative abundance of AOA was 42% in fraction II, which was the second peak. The abundance of AOB *amoA* gene also reached the maximum in fraction IV. Similar to sample A, the maximum peaks of AOA and AOB *amoA* gene abundance in sample B also appeared in fraction IV. The results indicated that both AOA and AOB were active in assimilating Na_2_^13^CO_3_.

## Discussion

GelGreen^™^ is a dimeric nucleic acid dye that forms a hairpin structure in the presence of nucleic acid and emits strong fluorescence [22], with an excitation wavelength of 488 nm and emission at 520 nm. Although GelGreen^™^ has been routinely used to stain DNA in agarose gels [23, 24], there are no available reports on its application in CsCl density gradients. Here, GelGreen^™^ was investigated as a replacement for SYBR^®^ Safe to stain DNA in CsCl density gradients. Compared to previous studies [2, 9] that used pure culture DNA, we used DNA directly extracted from environmental sample, allowing us to optimize protocols for environmental DNA-SIP assays. Results showed that 15X was the optimal concentration of GelGreen^™^ staining with detection limit of 0.2 μg environmental DNA.

Compared with SYBR^®^ Safe, GelGreen^™^ has a lot of advantages for staining DNA in CsCl density gradients. First, GelGreen^™^ was more sensitive than SYBR^®^ Safe as shown in Fig 3 where the fluorescence of DNA bands stained by GelGreen^™^ was stronger than those stained by SYBR^®^ Safe. Also, less than one-third the amount of SYBR^®^ Safe was needed for GelGreen^™^ to detect the same amount of DNA. Secondly, the cost of the usage of GelGreen^™^ was lower than SYBR^®^ Safe. Third, as a hairpin shaped dye when attached to DNA, GelGreen^™^ is non-fluorescent or minimally fluorescent by itself avoiding generation of background noise that could mask signals from target molecules [22] or if there is, it was completely negligible (Fig 3). Although SYBR^®^ Safe has been previously successfully used in environmental DNA staining in CsCl density gradients [9], the DNA bands stained by SYBR^®^ Safe in this study were too faint to be detected even if we strictly followed the recommendations of Martineau *et al*. [9]. In contrast, stable and strong DNA bands were observed when stained by GelGreen^™^, regardless of the concentrations or buffers used (TE or GB buffer).

Furthermore, since GelGreen^™^ only needed blue light for excitation, it avoided damages to the DNA band by not using UV irradiation, which then kept DNA with intact structure for downstream analysis. As shown in Fig 5, DNA bands became stronger with increasing dosage of environmental DNA. Although the detection limit of environmental DNA was 0.2 μg, we recommend the dosage of 0.5 μg.

For the retrieval of DNA from the gradients, the modified needle extraction procedure was shown to be efficient and effective, which overcame the disadvantages of traditional fractionation and needle extraction. For example, in traditional fractionation, it eliminates the use of syringe pump that is expensive and cannot prevent disturbance in the process retrieving the DNA. Also, the modified protocol allowed retrieval of samples even in low amounts and even prevented disturbance in the upper gradients, which are usually experienced using the needle extraction [2]. For the modified needle extraction procedure in this study, only needles and syringes were needed. Retrieval of DNA can be accurately and directly performed from top to bottom, which effectively prevented the gradients to be disturbed. The consistent observations in stained DNA bands and qPCR of bacterial 16S rRNA gene provide evidences for the reliability of GelGreen^™^ staining.

GelGreen^™^ was successfully applied to CsCl density gradient ultracentrifugation for environmental DNA-SIP assay. After ultracentrifugation, GelGreen^™^-stained DNA, which was extracted from the microorganisms incubated with ^13^C-labeled substrate, was separated into two DNA bands. Due to the high sensitivity of GelGreen^™^, both ^12^C-DNA bands and ^13^C-DNA bands were easily detected. The copy numbers of AOA and AOB *amoA* gene were also observed in high abundance in fraction IV, indicating that AOA and AOB were potentially responsible for the metabolism of Na_2_^13^CO_3_. These groups could be actively performing ammonia oxidation in the tested enriched activated sludge. This further demonstrates the usability of GelGreen^™^ in detecting labelled target DNA in environmental DNA using CsCl density gradient ultracentrifugation.

## Conclusion

GelGreen^™^ was investigated as an alternative to SYBR^®^ Safe as a stain in CsCl density gradients for DNA-SIP experiments. GelGreen^™^ was more sensitive than SYBR^®^ Safe regardless of the buffer used (TE or GB buffer). The background fluorescence of SYBR^®^ Safe masked the target DNA bands, but less or no background fluorescence was found in GelGreen^™^ stained samples. The optimal dosage of GelGreen^™^ was 8.85 μL (final concentration was 15X), which was approximately only one-third of the volume needed for SYBR^®^ Safe. Under this optimal dosage, the detection limit of environmental DNA with GelGreen^™^ was at 0.2 μg. The modified needle extraction we used has also effectively and efficiently retrieved the targeted DNA bands by fractionating CsCl density gradients just into four or five fractions, simplifying follow-up analyses. Stained by GelGreen^™^, DNA extracted from ^13^C-labeled enriched activated sludge formed two clear DNA bands after ultracentrifugation. Quantification of the 16S rRNA gene was consistent with those observed by staining the DNA bands, confirming the accuracy of GelGreen^™^ staining as applied in CsCl density gradients. This study concludes that GelGreen^™^ could be an excellent stain in CsCl density gradients for environmental DNA-SIP assays.
